# Urbanization is associated with modifications in DNA methylation in a small passerine bird

**DOI:** 10.1111/eva.13160

**Published:** 2020-11-13

**Authors:** Hannah Watson, Daniel Powell, Pablo Salmón, Arne Jacobs, Caroline Isaksson

**Affiliations:** ^1^ Evolutionary Ecology, Biology Department Lund University Lund Sweden; ^2^ Institute of Biodiversity, Animal Health and Comparative Medicine University of Glasgow Glasgow UK; ^3^ Global Change Ecology, School of Science, Technology and Engineering University of the Sunshine Coast Sippy Downs QLD Australia; ^4^ Department of Natural Resources Cornell University Ithaca NY USA

**Keywords:** birds, DNA methylation, epigenetics, RRBS, urbanization

## Abstract

Urbanization represents a fierce driver of phenotypic change, yet the molecular mechanisms underlying observed phenotypic patterns are poorly understood. Epigenetic changes are expected to facilitate more rapid adaption to changing or novel environments, such as our towns and cities, compared with slow changes in gene sequence. A comparison of liver and blood tissue from great tits *Parus major* originating from an urban and a forest site demonstrated that urbanization is associated with variation in genome‐wide patterns of DNA methylation. Combining reduced representation bisulphite sequencing with transcriptome data, we revealed habitat differences in DNA methylation patterns that suggest a regulated and coordinated response to the urban environment. In the liver, genomic sites that were differentially methylated between urban‐ and forest‐dwelling birds were over‐represented in regulatory regions of the genome and more likely to occur in expressed genes. DNA methylation levels were also inversely correlated with gene expression at transcription start sites. Furthermore, differentially methylated CpG sites, in liver, were over‐represented in pathways involved in (i) steroid biosynthesis, (ii) superoxide metabolism, (iii) secondary alcohol metabolism, (iv) chylomicron remodelling, (v) cholesterol transport, (vi) reactive oxygen species (ROS) metabolic process and (vii) epithelial cell proliferation. This corresponds with earlier studies identifying diet and exposure to ROS as two of the main drivers of divergence between organisms in urban and nonurban environments. Conversely, in blood, sites that were differentially methylated between urban‐ and forest‐dwelling birds were under‐represented in regulatory regions, more likely to occur in nonexpressed genes and not over‐represented in specific biological pathways. It remains to be determined whether diverging patterns of DNA methylation represent adaptive evolutionary responses and whether the conclusions can be more widely attributed to urbanization.

## 1 INTRODUCTION

The ability for organisms to adapt quickly is particularly pertinent in the context of current unprecedented rates of global environmental change (Rivkin et al., 2019; Schell, 2018). Urbanization—the process of formation and expansion of towns and cities—is proceeding at a phenomenal rate, across the world (United Nations, 2018), exerting novel selection pressures on free‐living organisms. Persistence of a species within the urban environment likely requires rapid adaptation to the wide range of novel environmental stressors to which animals find themselves subjected (Isaksson, 2015). Organisms living in the urban environment commonly show marked differences in a suite of phenotypic traits, relative to their countryside‐dwelling conspecifics (Cheptou et al., 2008; Chick et al., 2019; Lyons et al., 2017; Salmón et al., 2017; Slabbekoorn & Peet, 2003; Tennessen et al., 2014). However, to what extent variation at the phenotypic level, between urban and nonurban conspecifics, is driven by differences in genetic or nongenetic variation remains unclear.

While the process of genetic adaptation is usually slow and takes several generations to spread within a population, epigenetic modifications—broadly described as alterations to the structure of DNA—occur at a higher rate than changes to the DNA sequence. The epigenome is therefore expected to evolve faster than the genome, and epigenetic changes could be crucial in facilitating rapid adaptation in changing or novel environments, such as the world's growing towns and cities (Bossdorf et al., 2008; Hu et al., 2019; Hu & Barrett, 2017; Jablonka & Raz, 2009; Verhoeven et al., 2016). Indeed, epigenetic divergence has been shown to occur prior to genetic divergence following reproductive isolation in fish (Smith et al., 2016), and high epigenetic diversity, yet low genetic diversity, was found in an introduced population of a passerine bird (Liebl et al., 2013) and among closely related species of Darwin's finches (Skinner et al., 2014). Furthermore, accumulating evidence indicates that epigenetic marks can be induced or removed in response to environmental cues, within the lifetime of an individual (Bollati & Baccarelli, 2010; Gugger et al., 2016; Lea et al., 2016). Despite this suggested reversible plasticity, there is evidence that, if epigenetic modifications occur in early life, phenotypic traits can be permanently affected (Waterland & Jirtle, 2003; Weaver et al., 2004) with consequences for fitness (Rubenstein et al., 2016). Moreover, if environmentally induced epigenetic changes can be stably inherited across multiple generations, epigenetics has the potential to drive the evolutionary trajectory of populations in response to environmental change (Jablonka & Raz, 2009).

DNA methylation, involving the addition of a methyl group (‐CH3) to the 5' carbon of cytosines, is the most widely studied epigenetic mark and plays a key role in regulating gene expression and genome stability (Bird, 2002). Methylation of DNA primarily occurs at cytosine residues within CpG nucleotides, which are concentrated in gene regulatory regions (e.g. promoters and transcription start sites). Changes in DNA methylation can alter expression of associated genes, but the direction of the effect depends upon the genomic location. While methylation at CpG‐rich promoters is often associated with stable repression of gene expression, that is gene silencing, DNA methylation within the gene body has been shown to be positively correlated with gene expression in mammalian tissues (Jones, 2012; Lev Maor et al., 2015). Increasingly, DNA methylation patterns are being shown to be responsive to environmental cues, in wild (Kilvitis et al., 2019; Laubach et al., 2019; Lea et al., 2016) and captive (Jimeno et al., 2019; Viitaniemi et al., 2019) animal populations, suggesting a critical role for DNA methylation as a rapid regulator of phenotypic divergence and adaptation.

Genomic and epigenomic studies offer great potential to understand evolution in cities, yet they remain few and far between (Rivkin et al., 2019; Schell, 2018). While some studies have found genomic signatures of urban adaptation (Harris et al., 2013; Salmón et al., 2020) and consistent differences at the transcriptomic level (Watson et al., 2017), others have found no genetic structure among urban and nonurban congeners (Khimoun et al., 2020). Modifications in DNA methylation have been shown to associate with anthropogenic‐linked factors including feeding on anthropogenic‐provided food in baboons (Lea et al., 2016), exposure to pollution in humans (Baccarelli et al., 2009), and stress resilience in response to handling in tree swallows (Taff et al., 2019). A few studies have specifically investigated epigenetic variation in wild animals in the context of urbanization, revealing varied patterns (Garcia et al., 2019; McNew et al., 2017; Riyahi et al., 2015; Thorson et al., 2019).

In the present study, we investigated variation in genome‐wide DNA methylation of urban‐ and forest‐dwelling great tits *Parus major* with the aim of elucidating the potential for DNA methylation to mediate well‐established phenotypic differences between the two environments. Urban and nonurban populations of great tits have been shown to differ in respect of oxidative stress physiology (Salmón, Watson, et al., 2018), telomere dynamics (Salmón et al., 2016), immune function (Bailly et al., 2016), breeding performance (Sprau et al., 2017) and survival (Salmón et al., 2017), among other traits. In general, the urban environment appears to be more costly for great tits, but there is evidence for potential adaptation, with upregulation of expression of genes involved in protection against oxidative stress and immune function observed in our urban study population (Watson et al., 2017).

Using reduced representation bisulphite sequencing (RRBS), we characterized CpG methylation patterns in the liver and blood of great tits originating from an urban and a forest environment, and we investigated the functional relevance of observed environmentally induced differences in DNA methylation. Both the avian liver and blood have been shown to be sensitive to urbanization‐associated factors, such as changes in diet (Andersson et al., 2015; Møller et al., 2010) and pollutant exposure (Koivula & Eeva, 2010), and our previous research revealed concordant changes in gene expression in the two tissues, in response to urbanization (Watson et al., 2017). We predict that, if differences in DNA methylation patterns between urban‐ and forest‐dwelling birds represent regulated and coordinated responses associated with urbanization, CpG sites of differential methylation should be over‐represented in (i) regions of the genome regulating gene expression; (ii) regulatory regions associated with expressed, rather than nonexpressed genes; (iii) genomic regions involved in biological pathways relevant to response or adaptation to the urban environment (see below); and, (iv) methylation should correlate with expression of associated genes. Based on our previous findings (Salmón, Stroh et al., 2018; Salmón, Watson, et al., 2018; Watson et al., 2017), we predict that coordinated differences in DNA methylation would be associated with genes involved in immune function, protection against oxidative stress and lipid metabolism.

## 2 MATERIALS AND METHODS

### 2.1 Study species and sites

Twelve wild male great tits—six from an urban environment and six from a forest environment—were captured in late winter (February/March 2014) in southern Sweden. Urban birds were captured in urban parkland in Malmö (55°35'N 12°59'E)—the third largest city in Sweden with c. 300,000 inhabitants. Forest birds were captured in Vombs fure (55°39'N 13°33'E), a dense forest surrounded by areas of low human habitation (<5 inhabitants km^−2^) and located about 36 km east of the urban site. Biometrics were recorded, and a blood sample was collected and immediately transferred to storage at −80°C. Birds were subsequently euthanized, and postmortems were carried out immediately; liver tissue was collected and transferred to storage at −80°C within 5 min of death. All birds were adults: forest birds were all aged as 3 years or older; three of the urban birds were aged 2 years, while the other three were aged as 3 years or older. Due to high adult mortality (61%) and low recruitment rates (7%) at our sites and dispersal of great tit siblings (Dingemanse et al., 2003), genetic relatedness among birds within each habitat is expected to have minimal effect. Furthermore, genetic differentiation between the two sites is very low (F*_ST_* = 0.0055, (Salmón et al., 2020)), and all birds can be considered to originate from a single population.

### 2.2 DNA isolation, bisulphite treatment and sequencing

DNA was isolated from 5 µl whole blood and 25 mg liver tissue using NucleoSpin Blood and Tissues kits (Macherey‐Nagel, Germany), respectively, and according to the manufacturer's protocol. Sample quality was assessed using ultraviolet spectrophotometry (NanoDrop, Thermo Scientific) and gel electrophoresis. Average (median ± *SD*) absorption ratios were 1.95 ± 0.08 (260/280) and 2.57 ± 0.13 (260/230). RRBS library preparation and sequencing were carried out by the SNP&SEQ technology platform (Sweden). RRBS libraries were prepared using 270 ng DNA and an in‐house protocol; DNA was cut using the *Msp*I restriction enzyme and then subjected to bisulphite treatment. Paired‐end (125 bp read length) sequencing was performed using the Illumina HiSeq 2,500 platform with high output mode, v4 chemistry and 12 samples per lane. A PhiX spike‐in of 30% was included.

### 2.3 Reduced representation bisulphite sequencing sequence alignment and methylation calling

Raw reads are deposited in NCBI's Sequence Read Archive (PRJNA314210, NCBI BioProject, 2017). Raw reads were processed for adapter trimming and removal of low‐quality reads and artificially added bases using Trim Galore! 0.4.4 (Krueger, 2015) with the parameters ‐‐rrbs ‐‐paired ‐‐max_n 1 ‐‐quality 25 ‐‐length 40 ‐‐clip_R2 5. Quality filtering resulted in an average (mean ± *SE*) of 16.48 ± 2.06 million and 19.94 ± 2.16 million high‐quality paired‐end reads from liver and blood tissues, respectively, per individual. We tested for differences in the total number of CpG sites sequenced (*n* = 6 birds/habitat; unpaired) using a two‐sample Wilcoxon signed‐rank test in R 3.6.1 (R Core Team, 2013). Trimmed reads were aligned to the great tit genome (Laine et al., 2016) (assembly: Parus_major1.1; RefSeq accession: GCF_001522545.2) using Bismark 0.18.2 (Krueger & Andrews, 2011) in the mode for paired‐end nondirectional RRBS data, including the parameters ‐‐bowtie2 ‐‐fastq ‐X 1,000 ‐‐score_min L,0,‐0.6. An average of 54.1% (liver) and 54.2% (blood) reads aligned uniquely, consistent with other reports (Chatterjee et al., 2012; Yuan et al., 2016). Methylation calling was performed using the bismark_methylation_extractor tool with the parameters ‐‐paired‐end ‐‐no_overlap ‐‐ignore_r2 2. Coverage files were analysed in methylKit (Akalin et al., 2012) to call differentially methylated sites (DMSs) between urban and forest birds, merging reads on both strands of a CpG dinucleotide, including only CpG sites covered in all samples, and discarding bases that had a read coverage lower than ten and with more than the 99.9th percentile coverage in each sample. CpG sites were considered differentially methylated (and thus associated with urbanization) with a methylation difference larger than 25% and a *q*‐value <0.01 in line with other recent studies (Aluru et al., 2018; McCormick et al., 2017). *P*‐values were adjusted to *q*‐values using the SLIM method (Wang et al., 2011). Permutation tests were performed by carrying out the differential methylation analysis, with 1,000 iterations, each time permuting habitat assignments of individuals; the observed differences in DNA methylation were compared to the permutation distribution.

### 2.4 RNA‐seq alignment

RNA sequencing of the liver and blood transcriptomes from the same 12 individuals was carried out for an earlier study (Watson et al., 2017), and raw reads are deposited in NCBI's Sequence Read Archive (PRJNA314210, NCBI BioProject, 2017). Read quality was first appraised using FastQC 0.11.5 software (Andrews, 2010), and quality trimming and filtering were applied to remove low‐quality reads and adaptor contamination in Trimmomatic (Bolger et al., 2014). Clean reads were aligned to annotated regions of the great tit genome (see above) using HISAT 2.1.0 (Kim et al., 2015) and sorted with SAMtools 1.5 (Li et al., 2009). Counts were made using StringTie 1.3.3 (Pertea et al., 2015) in accordance with the protocol described by Pertea et al. (2016). Genes were classified as being expressed if a read count of ten or higher was found in three or more (≥25%) individuals. This represented a slightly more conservative threshold than used by Lea et al. (2016) with a bigger sample size.

### 2.5 Genomic locations and gene annotation

In order to evaluate the distribution of sequenced CpG sites throughout the genome, coverage thresholds were relaxed to a minimum of 3 reads in at least one individual. CpG sites were assigned to genomic locations and genes using annotated data from the great tit genome. Sites were assigned to either a) gene body, b) promoter, c) transcription start site (TSS), d) CpG island (CGIs), e) CpG island shore or f) nonregulatory region. Promoters were defined as the region 3 kb upstream from gene start (excluding TSS); TSS regions were defined as the region from 300 bp upstream and 50 bp downstream of gene start; and, CGIs were defined as regions where, over an average of 10 windows of 100 bp and a minimum of 200 bases, the calculated GC content is>=50% and the ratio of observed to expected GC dinucleotides is 0.6, all as per Viitaniemi et al. (2019). CpG island shores were defined as 2 kb regions flanking CpG islands (Irizarry et al., 2009; Rao et al., 2013). Overlap between CpG sites and regulatory regions was determined by intersection using BEDtools 2.27.1 (Quinlan & Hall, 2010), and CpG sites were assigned to one or more specific genes, where overlaps were identified. Prior to the assignment, overlapping regions annotated to the same gene were merged.

### 2.6 Over‐representation of differentially methylated sites

If changes in DNA methylation associated with urbanization represent coordinated responses to the environment, it is expected that differences in methylation should (i) occur in regions of the genome regulating gene expression; (ii) occur in regulatory regions associated with expressed, rather than nonexpressed genes; (iii) be concentrated in genomic regions involved in related biological pathways; and (iv) relate to changes in gene expression of associated genes. We addressed each of these predictions in turn, as described below. All analyses were performed in R 3.6.1 (R Core Team, 2013) using DMSs as called in the pipeline described above.

Using Fisher's exact test, we tested whether (i) DMSs are over‐represented in functional (i.e. regulatory) regions of the genome and under‐represented in regions with no regulatory function, and (ii) DMSs are more likely to occur in expressed genes. Since RRBS enriches for likely functional areas of the genome, the sequenced CpG sites are expected to be over‐representative of these functional genomic regions. Over‐representation was therefore assessed relative to the background of all sequenced CpG sites. The existence of multiple DMSs in close proximity to one another is more likely to be of functional relevance for gene expression (Lister et al., 2009). We therefore also tested for over‐representation of urbanization‐linked sites in expressed genes, by considering only those DMSs occurring within “differentially methylated regions” (DMRs), defined as the occurrence of three or more urbanization‐linked sites within a 2 kb window, following Lister et al. (2009). For the assessment of over‐representation of DMRs in expressed genes, the background dataset similarly comprised only CpG sites that were located within 2 kb of two or more other CpG sites.

To investigate the functional relevance of differences in DNA methylation, we performed gene over‐representation analyses to test whether (iii) urbanization‐linked CpG sites are over‐represented in or near genes involved in related and relevant biological pathways. Over‐representation analyses were carried out using the BiNGO 3.0.2 plug‐in in Cytoscape 3.2.1, using the great tit gene models as the background. Given the expectation that DMRs are of greater functional relevance, we included only those genes associated with DMSs located within a DMR (defined above) and overlapping with either the gene body, promoter or TSS, using the annotation described above. A full ontology file was created using the annotated great tit genome and the core Gene Ontology (GO) downloaded from www.geneontology.org on 13 September 2018. Hypergeometric tests were employed, and *P*‐values were corrected using the Benjamini and Hochberg false discovery rate (FDR) (Benjamini & Hochberg, 1995). GO terms with FDR < 0.05 were considered to be significantly over‐represented. GO terms were summarized and redundant terms eliminated, using a clustering algorithm based on semantic similarity in Revigo (Supek et al., 2011), and over‐represented terms were visualized in CirGO (Kuznetsova et al., 2019).

Lastly, we tested whether (iv) DNA methylation at individual DMSs is correlated with gene expression, where DMSs were located in the gene body, TSS or promoter region associated with a gene for which expression levels were also quantified. Linear mixed models were fitted to data on gene expression counts with the covariate of methylation level, the fixed factor of feature (three levels: gene body, TSS or promoter) and the interaction between methylation and feature. A random effect of individual was included, though the estimated variance was zero. Prior to analysis, read expression counts were rlog‐transformed in DESeq2 (Love et al., 2014). Models were fitted to data from liver and blood separately. Pairwise comparisons of slopes were carried out using emtrends() and the Tukey method for *P*‐value adjustment.

## 3 RESULTS

### 3.1 Urbanization influences genome‐wide DNA methylation

DNA methylation was quantified at 6,166,831 and 5,130,570 CpG sites across the great tit genome, in liver and blood, respectively. Average (mean ± *SE*) read coverage was 8.7 ± 0.04 and 15.7 ± 0.2 across all analysed CpG sites in liver and blood, respectively. There were no consistent differences in the number of CpG sites sequenced in urban birds (*n* = 6; mean ± *SE*: liver: 3,645,356 ± 87,634; blood: 3,654,552 ± 42,805), compared with forest birds (*n* = 6; mean ± *SE*: liver: 3,513,949 ± 109,839; blood: 3,641,601 ± 38,057) in either liver (*W* = 22, *p* = .6) or blood (*W* = 16, *p* = .8). A majority of CpG sites were hypomethylated (i.e. median methylation < 50%) in both liver (68.2%) and blood (66.8%). Most CpG sites were methylated at low levels (median ± *SD*: liver = 11.8 ± 29.4%; blood = 9.09 ± 33.3%), but average methylation ranged from completely unmethylated to constitutively (100%) methylated.

**Figure 1:**
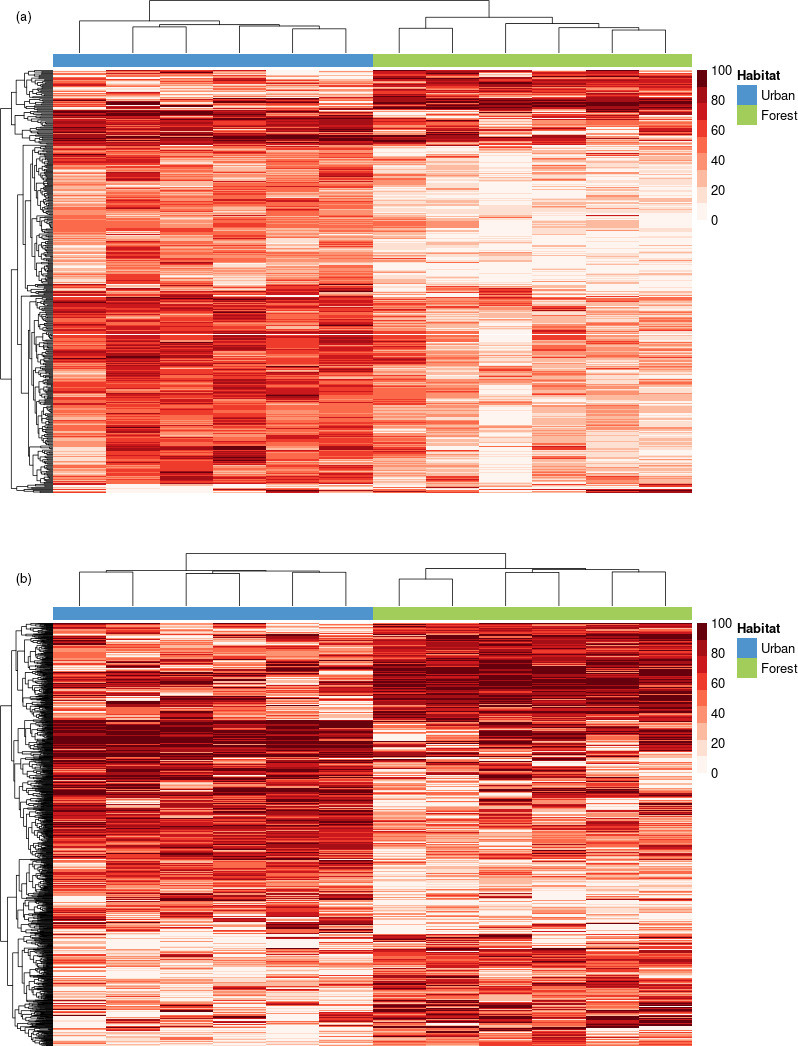
Hierarchical clustering of differentially methylated sites in urban‐ and forest‐dwelling great tits *Parus major*in liver (a) and blood (b). Each column represents an individual from either the urban (blue) or forest (green) habitat. Each row represents a CpG site at which DNA methylation levels were significantly different between the two habitats. The colour scale represents percent DNA methylation, with higher methylation indicated by a darker shade of red

A total of 419 (liver) and 1,144 (blood) CpG sites were differentially methylated between urban and forest birds, and thus from hereon, we refer to these CpG sites and the observed differences in their DNA methylation levels, as being linked to or associated with urbanization (Figure 1). Average (mean ± *SE*) read coverage at DMSs was 33.3 ± 1.3 and 34.1 ± 0.6 in liver and blood, respectively. Permutation tests revealed that the observed differences in DNA methylation were significantly different to that expected by chance (liver: *p* = .002; blood: *p* = .01). Of the differentially methylated sites, in liver, the majority (88.5%) were hypermethylated in urban, compared with forest, birds. In blood, similar proportions of urbanization‐linked CpG sites were hypomethylated (49.3%) and hypermethylated (50.7%) in urban, compared with forest, birds. In liver, 19 DMRs (defined as three or more DMSs within a 2 kb window) were identified, involving 102 DMSs; seventeen (89.5%) DMRs exhibited constitutive hypermethylation (across the constituent DMSs) in urban, compared with forest, birds. A particularly large DMR was identified on the Z chromosome in liver tissue, comprising a total of 36 urbanization‐linked CpG sites within a 300 bp window, but none of the CpG sites were associated with any annotated genes. In blood, 54 DMRs were identified, involving 226 DMSs; eighteen DMRs (33.3%) were constitutively hypomethylated and 14 (25.9%) were constitutively hypermethylated.

### 3.2 Urbanization‐linked sites are over‐represented in certain genomic regions

**Figure 2:**
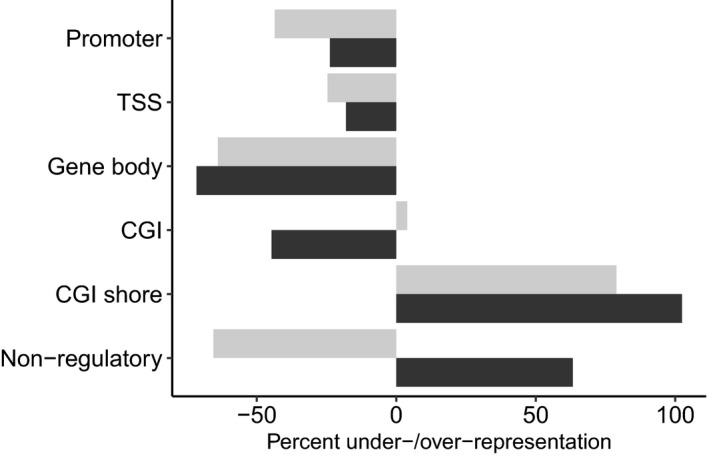
CpG sites associated with urbanization were distributed nonrandomly throughout the genome of great tits*Parus major*. Under‐/over‐representation of urbanization‐associated sites in various regulatory and nonregulatory regions of the genome is illustrated in the liver (grey) and blood (black). Urbanization‐linked differences in DNA methylation were under‐represented in the regulatory regions of promoters, transcription start sites (TSS), gene bodies and CpG islands (CGI; blood only), but were more likely to occur within CGI shores, the regulatory regions flanking CpG islands. Differentially methylated sites were under‐represented in nonregulatory regions in liver, but over‐represented in un‐annotated regions in blood

In accordance with the fact that RRBS enriches for likely functional areas of the genome, a large proportion of sequenced CpG sites occurred within regulatory genomic regions (Table 1; liver: 88.5%, blood: 91.8%). Overall, urbanization‐associated CpG sites were over‐represented in regulatory regions of the genome, in liver (odds ratio = 1.5, *p* = .009), but they were under‐represented in regulatory regions in blood (odds ratio = 0.61, *p* < .001; Table 1; Figure 2). Specifically, DMSs were less likely to occur within a promoter, TSS, gene body (all *p* < .001) or CGI (blood only: *p* < .001), but were more likely to be located within CGI shores, the regulatory regions flanking CpG islands in both liver (odds ratio = 1.8, *p* < .001) and blood (odds ratio = 2.02, *p* < .001). In liver, there was no difference in the frequency of occurrence of CpG sites in CGIs between the DMS and background data sets (odds ratio = 1.04, *p* = .7). DMSs were associated with 220 unique genes in liver and 492 unique genes in blood.

**Table 1:** Summary of genomic locations of all sequenced CpG sites and differentially methylated sites (DMSs) between urban and forest great tits in liver and blood*

	Total	Promoter	TSS	Gene body	CGI	CGI shore**	Nonregulatory
Liver							
All CpG	6,166,831	2,440,376 (39.6%)	1,508,486 (25.5%)	3,961,469 (64.2%)	3,002,199 (48.7%)	1,316,950 (21.4%)	711,340 (11.5%)
DMSs	419	93 (22.2%)	31 (7.4%)	224 (53.5%)	208 (49.6%)	137 (32.7%)	33 (7.9%)
Blood							
All CpG	5,130,570	2,197,364 (42.8%)	1,346,853 (26.3%)	3,339,042 (65.1%)	2,882,780 (56.2%)	1,148,229 (22.4%)	421,862 (8.2%)
DMSs	1,144	173 (15.1%)	69 (6.0%)	654 (57.2%)	417 (36.5%)	422 (36.9%)	146 (12.8%)

Note

Coverage thresholds adopted were 3x for all CpG sites and 10x for DMSs.

Abbreviations: CGI, CpG island; TSS, transcription start site.

* Note that CpG sites can be associated with one of more genomic features.

** CGI shores are defined as 2 kb regions flanking CpG islands.

Urbanization‐linked CpG sites were not more likely to occur in regulatory regions associated with expressed genes, compared with nonexpressed genes, in either liver (odds ratio = 0.99, *p* = .6) or blood (odds ratio = 0.56, *p* = 1). In fact, DMSs in blood were more likely (*p* < .001) to occur in or close to genes that were not expressed (37.5%), compared with the background CpG sites (25.1%). When considering only those differentially methylated sites located within DMRs, in liver, all sites (100%) were located within regulatory regions associated with expressed genes, and this was significantly more so than in the background data set (84.6%; odds ratio = inf.; *p* < .001). In blood, DMSs located within DMRs were again less likely to be located in regions associated with expressed genes (68.1%), compared with the background (75.0%; odds ratio = 0.71; *p* = .04).

### 3.3 Functional relevance of urbanization‐associated sites

**Figure 3:**
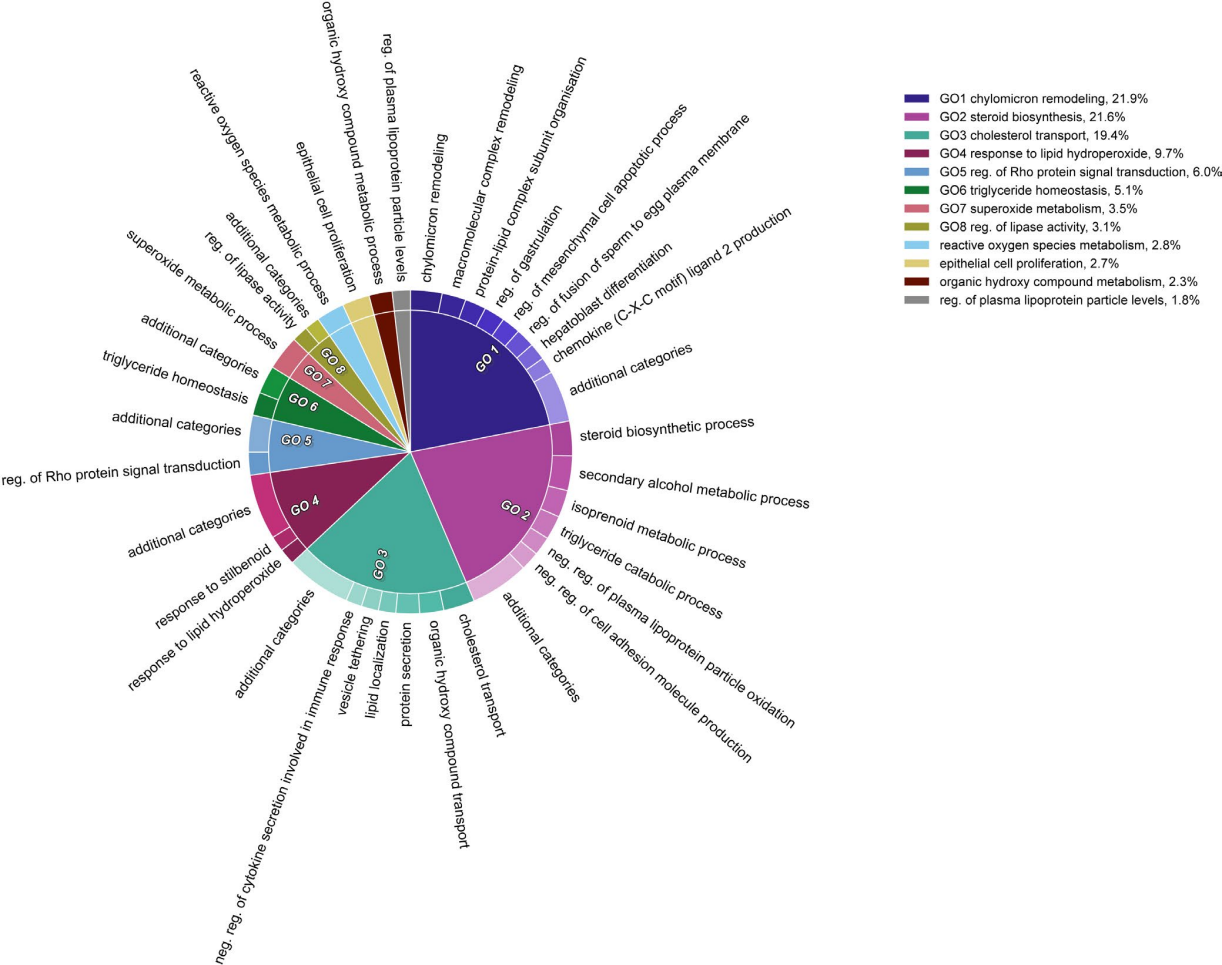
Representation of functional role of genomic regions that are differentially methylated between urban‐ and forest‐dwelling great tits *Parus major*, in liver tissue. Nonredundant over‐represented gene ontology (GO) terms are grouped by hierarchical clustering. Parent terms are identified in the legend along with their respective proportions, which are directly proportional to statistical significance. GO terms were first summarized based on a semantic similarity of 0.4 using REVIGO and visualized in CirGO. Abbreviations: neg., negative; pos., positive; reg., regulation

Over‐representation analyses revealed that urbanization‐associated regions, in liver, were over‐represented in a few related biological pathways, but there was no such over‐representation among differentially methylated regions in blood. Sixteen genes associated with DMRs in liver and with ontology annotation (a further four genes had no ontology information available) matched to 1,109 GO terms. Of the associated GO terms, 206 terms were significantly over‐represented at an FDR of 0.05. In blood, 26 (out of 34) genes associated with DMRs had available ontology annotation and matched to 1,037 GO terms, but none of the terms were over‐represented at an FDR level of either 0.05 or 0.1. Elimination of redundant GO terms associated with DMRs in liver tissue, using a semantic similarity threshold of 0.4, yielded a subset of 30 representative GO terms, providing a succinct summary of over‐represented pathways (Table 1; Figure 3). The GO terms of highest significance (FDR ≤  0.002) and lowest dispensability (<0.1) were as follows: (i) steroid biosynthetic process, (ii) superoxide metabolic process, (iii) secondary alcohol metabolic process, (iv) chylomicron remodelling, (v) cholesterol transport, (vi) reactive oxygen species metabolic process and (vii) epithelial cell proliferation.

We identified seven genes associated with urbanization‐linked CpG sites and whose functions are linked to the most significant over‐represented pathways in the gene ontology network, as highlighted above. These include three genes with key roles in cholesterol transport and metabolism: the apolipoprotein encoded by APOA1 modulates reverse transport of cholesterol from tissues to liver; APOA4 encodes an apolipoprotein that forms a component of chylomicrons and high‐density lipoproteins; and STARD3 encodes a sterol‐binding protein that also mediates cholesterol transport. Another gene, HNF1B, is also linked to cholesterol transport via its involvement in secretion by cells, as well as being linked to epithelial cell proliferation. Farnesyl pyrophosphate synthase, encoded by FDPS, catalyses a pathway upstream of many essential metabolites including sterols and carotenoids. NOX5 and NOXA1 are major sources of superoxide generation and involved in the regulation of redox‐dependent processes. Each of these genes has a cluster of between three and five differentially methylated sites associated with the gene body, promoter and/or TSS region. All, except one, of the involved CpG sites are hypermethylated in urban, compared with forest, birds.

### 3.4 DNA methylation is linked to gene expression

**Figure 4:**
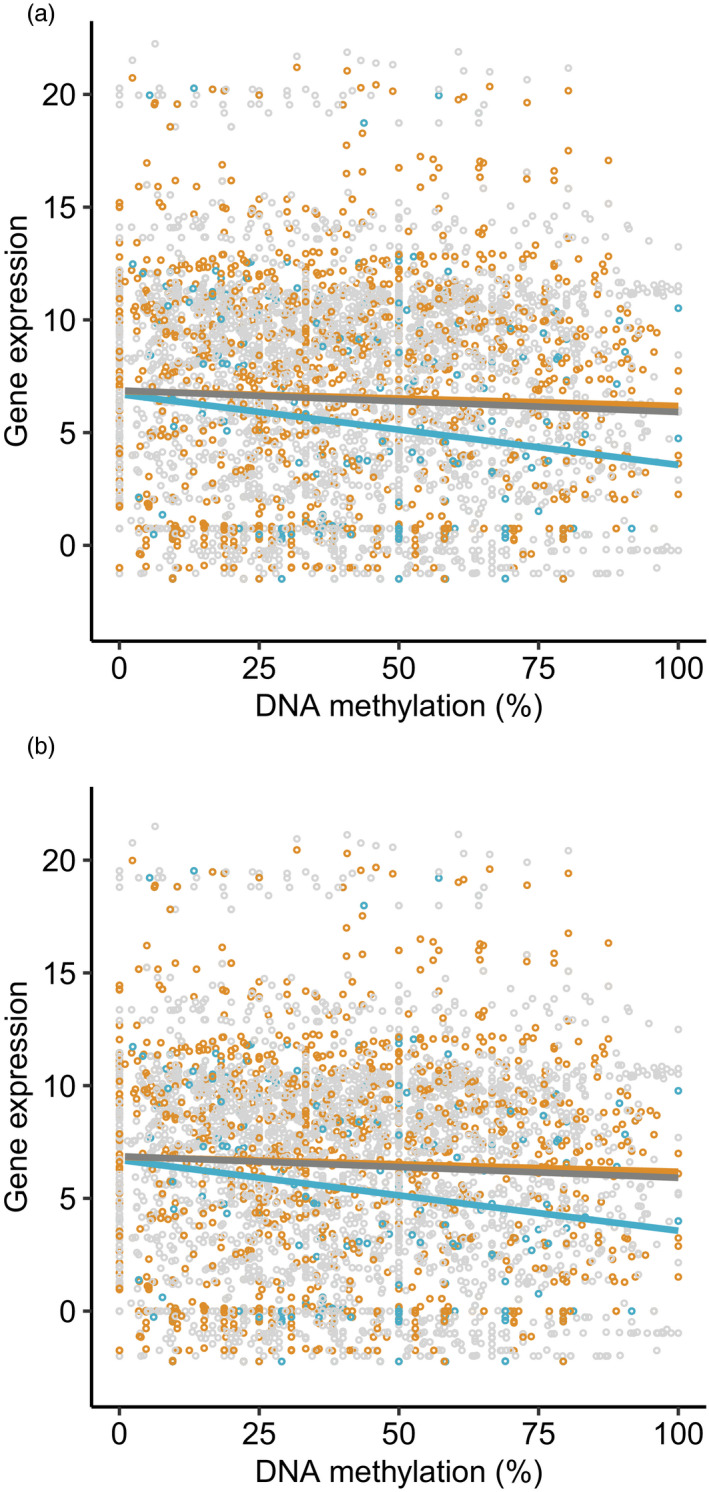
Relationship between gene expression (rlog‐transformed read count) and DNA methylation (%) of urbanization‐associated CpG sites located in gene bodies (grey), promoters (orange) and transcription start sites (blue; TSS) of respective genes in (a) liver and (b) blood of great tits *Parus major* (*n* = 12). Lines represent fitted lines from linear models including the interaction between DNA methylation and genomic feature. The slope of the relationship between gene expression and DNA methylation at TSSs was significantly different to zero and different to that of genes or promoters, but there is a large amount of residual variation in expression

Significant variation in gene expression was explained by variation in methylation level at associated DMSs, but the slope of the correlation depended on whether an urbanization‐associated CpG site was located in the gene, promoter or TSS region, in both liver (methylation*feature: *F*_2,4,842_ = 2.93, *p* = .05; Figure 4a) and blood (methylation*feature: *F*_2,12,738_ = 3.32, *p* = .04; Figure 4b). Post hoc tests confirmed that gene expression was negatively correlated with DNA methylation when a DMS was located in a TSS in liver (slope = −3.1e^−2^, 95% CI = −4.9e^−2^‐−1.3e^−2^) and blood (slope = −1.1e^−2^, 95% CI = −2.0e^−2^‐‐3.3e^−3^), and the slopes were either significantly different, or close to being significantly different, from that of either gene body (liver: *p* = .07; blood: *p* = .05) or promoter (liver: *p* = .05, blood: *p* = .04) regions. Expression was also negatively correlated with methylation in gene bodies in liver (slope = −9.6e^−3^, 95% CI = −1.6e^−2^‐‐2.7e^−3^). The estimated slopes of the relationship between expression and methylation were not significantly different to zero, when a DMS was located in the promoter in both tissues (liver: slope = −6.6^e3^, 95% CI = −1.7e^−2^–3.2e^−3^; blood: slope = 1.0e^−3^, 95% CI = −4.7e^−3^_–_–6.7e^‐3^) and when a DMS was located in a gene body in blood (slope = −9.3e^−4^, 95% CI = −4.1e^−3^–2.2e^−3^). The slopes for gene and promoter regions were not significantly different from one another (liver: *p* = .9; blood: *p* = .8). A large amount of residual variation in gene expression levels remained, which was not explained by variation in methylation.

## 4 DISCUSSION

We present one of the first studies to examine DNA methylation patterns in the context of urbanization in vertebrates (Riyahi et al., 2015; McNew et al., 2017; though see Garcia et al., 2019), which provides important advances to our understanding of the role of epigenetic modifications in mediating ecologically relevant phenotypic changes and thereby adaptation. Our results reveal variation in DNA methylation, in both the liver and blood of wild great tits, that corresponds with habitat differences associated with urbanization. Diet and ROS exposure are pinpointed as the possible main drivers of epigenetic divergence between the two populations, based on the biological pathways associated with differences in DNA methylation patterns in the liver. In turn, this suggests that the observed variation in DNA methylation could be driven by variation in food availability and/or pollutant levels, both of which have previously been associated with divergence in DNA methylation patterns (Lea et al., 2016; Romano et al., 2016) and are well understood to characterize key differences between urban and nonurban areas. Furthermore, the results are in accordance with previous studies highlighting marked differences in phenotypic traits, especially those linked to diet and oxidative stress, between urban and nonurban animal populations (Costantini et al., 2014; Isaksson et al., 2007, 2009). However, it is important to acknowledge that, in the absence of replicated sampling sites, we cannot be certain that the results would be reproduced across other populations or that likely causal factors are explicitly a consequence of urbanization rather than other site‐specific differences. Alternatively, variation in DNA methylation could arise as a result of nonadaptive genetic variation between urban and nonurban populations (e.g. drift or ancestry (Husquin et al., 2018)). Further studies are required to determine whether the results can be more widely attributed to urbanization and whether the observed divergent patterns of DNA methylation represent an adaptive evolutionary response.

In line with our predictions associated with a coordinated environmentally induced response, urbanization‐associated CpG sites of differential methylation in liver were (i) over‐represented in regulatory regions of the genome (Figure 2), (ii) more likely to occur in association with expressed genes, (iii) concentrated in relevant biological pathways (Table 2, Figure 3), and (iv) related to gene expression in a manner that was dependent on genomic feature (Figure 4), consistent with other studies (Derks et al., 2016; Jones, 2012). Furthermore, we found almost ubiquitous hypermethylation in the livers of urban, compared with forest, birds, which provides further evidence for a coordinated environmental response (Figure 1). Interestingly, the same pattern of higher levels of DNA methylation in urban birds, relative to rural birds, was also found in two candidate genes linked to personality in great tits (Riyahi et al., 2015).

**Table 2:** Functional roles linked to genomic regions that are differentially methylated between urban‐ and forest‐dwelling birds, in liver tissue

GO ID	GO description	Size	log10 *p*‐value	Nested terms
GO:0006694	Steroid biosynthetic process	0.12	−3.31	33
GO:0006801	Superoxide metabolic process	0.14	−3.31	9
GO:0034371	Chylomicron remodelling	0.00	−3.00	13
GO:1901615	Organic hydroxy compound metabolic process	0.83	−2.20	0
GO:0042157	Lipoprotein metabolic process	0.21	−1.46	0
GO:0072593	Reactive oxygen species metabolic process	0.28	−2.67	0
GO:0030301	Cholesterol transport	0.02	−2.97	19
GO:0050673	Epithelial cell proliferation	0.07	−2.62	1
GO:0070328	Triglyceride homeostasis	0.01	−2.20	7
GO:1902652	Secondary alcohol metabolic process	0.10	−3.31	3
GO:0034445	Negative regulation of plasma lipoprotein particle oxidation	0.00	−1.79	1
GO:0035023	Regulation of Rho protein signal transduction	0.13	−2.20	5
GO:0060191	Regulation of lipase activity	0.01	−1.53	5
GO:0097006	Regulation of plasma lipoprotein particle levels	0.01	−1.74	0
GO:0099022	Vesicle tethering	0.00	−1.63	1
GO:0006982	Response to lipid hydroperoxide	0.00	−1.53	2
GO:0071702	Organic substance transport	4.98	−1.35	2
GO:0002227	Innate immune response in mucosa	0.00	−1.43	5
GO:0034367	Macromolecular complex remodelling	0.01	−2.28	0
GO:0060354	Negative regulation of cell adhesion molecule production	0.00	−1.79	1
GO:0010470	Regulation of gastrulation	0.01	−1.99	55
GO:0015850	Organic hydroxy compound transport	0.08	−2.29	1
GO:0061017	Hepatoblast differentiation	0.00	−1.63	2
GO:0072567	Chemokine (C‐X‐C motif) ligand 2 production	0.00	−1.53	20
GO:0014012	Peripheral nervous system axon regeneration	0.00	−1.43	2
GO:0033384	Geranyl diphosphate biosynthetic process	0.00	−1.64	1
GO:0071825	Protein–lipid complex subunit organization	0.01	−2.02	3
GO:0043012	Regulation of fusion of sperm to egg plasma membrane	0.00	−1.79	1
GO:0019433	Triglyceride catabolic process	0.01	−2.29	11
GO:0035634	Response to stilbenoid	0.00	−1.47	0

Note

Significantly (FDR ≤ 0.05) over‐represented gene ontology (GO) terms were summarized based on a semantic similarity of 0.4. Cluster representatives are listed in order of increasing dispensability (i.e. semantic similarity), along with their size, based on the frequency of occurrence in the background GO data (higher frequency = more general terms; lower frequency = more specific terms); adjusted *p*‐value; and, number of significant GO terms nested within each cluster.

It could be that the urban environment—with its novel stressors or altered resource base—selects for greater phenotypic plasticity, which could be mediated to some extent by DNA methylation. Given the accumulating evidence that conditions experienced during development are crucial in inducing stable phenotypic traits via epigenetic modifications (Jablonka & Raz, 2009; Weaver et al., 2004), it could be that the differences in DNA methylation observed in the present study are established during early life. Indeed, it has been demonstrated that growing up in the urban environment has marked consequences for survival and fitness‐related traits in birds (Salmón et al., 2017; Salmón, Watson, et al. 2018; Sprau et al., 2017), and furthermore, the early life environment has been shown to be a key determinant of DNA methylation patterns in the wild (Laubach et al., 2019; Rubenstein et al., 2016). A key aim for future studies of environmentally induced changes in DNA methylation should be to attempt to disentangle the relative contribution of genetic and environmental variation during the critical period of early life.

While there were differences in DNA methylation patterns in the blood, associated with urbanization, there was little evidence that this reflected a coordinated organismal response to environmental cues: sites of differential methylation between the two habitats were (i) not over‐represented in regulatory regions, (ii) less likely to occur in association with expressed genes, and (iii) not over‐represented in specific biological pathways; however, as in liver, DNA methylation in blood was negatively related to gene expression when DMSs were located in TSS regions. Although other studies have found DNA methylation patterns in blood to reflect environmental and social factors (Jimeno et al., 2019; Lea et al., 2016), tissue‐specific patterns of DNA methylation have been shown in mammals (Christensen et al., 2009) and birds (Siller & Rubenstein, 2019), and it is therefore not expected that all tissues will respond in the same way to environmental cues.

Differentially methylated sites were over‐represented in CGI shores, in both the liver and blood. While it has traditionally been considered that methylation at CGIs themselves is of greatest functional significance, increasing evidence suggests a key role for DNA methylation in the region of CGI shores. It has been recently shown that methylation of DNA in the regions flanking CGIs is more dynamic and variable than that within CGIs themselves, and changes in these regions appear to contribute to cancer development and tissue differentiation (Doi et al., 2009; Irizarry et al., 2009; Rao et al., 2013). Furthermore, Irizarry and colleagues (2009) demonstrated that differential DNA methylation of CGI shores correlates well with tissue‐specific gene expression. The accumulating evidence thus suggests a key involvement for CGI shore methylation in adaptive epigenetic programming. While other studies in the wild have shown environmentally induced differential methylation of DNA to be over‐represented in regions better known to be associated with gene expression (e.g. gene bodies, promoters and TSS) (Gugger et al., 2016; Lea et al., 2016), our observed over‐representation of urbanization‐associated sites in CGI shores could be equally important in regulating gene expression and phenotypic traits. Further research is needed to better understand the role of CGI shore methylation.

The biological pathways with which urbanization‐linked CpG sites were associated, in liver, pinpoint diet and ROS exposure as the two major drivers of the divergence between the urban‐ and forest‐dwelling birds. This is corroborated by previous findings at the transcriptomic level in the liver, where diet and oxidative stress were also identified as key processes underlying differences in gene expression between the same urban and forest populations (Watson et al., 2017). However, transcriptional differences in immune‐related genes, found in that study, were not matched by differences in DNA methylation. Over‐representation of genes, associated with differentially methylated regions, in pathways involved in lipid metabolism and transport suggests differences in energetic demands and/or dietary intake between the urban and forest birds, which could reflect differences in thermoregulatory requirements and resource availability, respectively. Indeed, differences in fatty acids circulating in the plasma have previously been shown in our study populations (Andersson et al., 2015). It is often assumed that urban‐dwelling birds rely more on anthropogenic‐provided foods, which have a higher fat content and different composition of fatty acids (Andersson et al., 2018), compared with naturally available foods (Gavett & Wakeley, 1986; Isaksson et al., 2014). Differential intake of dietary lipids could thus underlie the observed differences in DNA methylation patterns. Since the liver is a major site of lipid biosynthesis and metabolism, and transport of cholesterol and lipoproteins to and from other tissues, we would not necessarily expect to see the same patterns in the blood.

Metabolic processes involving ROS, which include the highly reactive superoxide and peroxides, are essential in the regulation of pro‐oxidants and protection against oxidative stress‐induced damage in the cells of the body. Oxidative stress describes a pathological state that arises when either a surplus of pro‐oxidants or depletion of antioxidants causes an increase in oxidative damage of DNA, lipids and/or proteins. Oxidative stress is considered to be almost ubiquitous in underlying the negative effects of many pollutants and stressors. The urban environment abounds with pro‐oxidant‐generating pollutants, and pollutant‐induced oxidative stress has been demonstrated in urban birds (Fossi et al., 1991; Isaksson, 2010). In turn, increased oxidative stress could be the driver of accelerated erosion of telomeres (Reichert & Stier, 2017), as has been observed in urban bird nestlings (Salmón et al., 2016). However, higher circulating levels of antioxidants (Salmón, Stroh et al. 2018) and elevated expression of several genes involved in metal detoxification and DNA repair (Watson et al., 2017), previously observed in our urban population, could indicate regulated responses to enable birds to cope with living in an environment rich in pro‐oxidants. The over‐representation of differentially methylated regions in genes associated with the metabolism of ROS provides further evidence for the importance of exposure to ROS in shaping the differences between birds living in cities and those living in more benign nonurban environments.

If variation in DNA methylation is mechanistically linked to variation at the phenotypic level, it is expected that there should be effects on regulation of gene expression. However, while often assumed, correlations between DNA methylation and gene expression are commonly not found in practice. It is now becoming increasingly understood that the functional relationship between CpG methylation and gene expression is not consistent across the genome (Lea et al., 2018), or even between CpG sites within a single promoter, as recently demonstrated in birds (Jimeno et al., 2019). Furthermore, additional mechanisms, including non‐CpG methylation and histone acetylation, influence transcription (Derks et al., 2016; Weaver et al., 2004). By using a reduced representation technique, we only sequenced a subsample of the genome's CpG sites. It is therefore perhaps not surprising that our results revealed relatively weak relationships between DNA methylation and gene expression, with large amounts of unexplained variation. This does not mean that there is no functional relevance to the observed variation in DNA methylation patterns, but that the nature and strength of associations are not consistent and/or are not understood in full because the whole methylome has not been sequenced.

Collectively, the observed results suggest that epigenetic mechanisms—specifically DNA methylation—could play a key role in regulating organismal responses to novel environments, such as towns and cities. Observed variation in DNA methylation corresponded with habitat differences associated with urbanization. In liver, CpG sites that were differentially methylated between the two habitats were over‐represented in regulatory regions of the genome, in expressed genes, and in biological pathways that pinpoint diet and oxidative stress as key drivers shaping differences between the urban‐ and forest‐dwelling birds. Conversely, we found little evidence for a coordinated environmental response in DNA methylation patterns in blood. Whether these patterns are consistent across other urban and forest populations, and thus can be more widely attributed to the process of urbanization, remains to be confirmed. An aim for the field of ecological epigenetics is to now further our understanding of the causal mechanisms, and consequences for fitness, of environmentally induced changes in DNA methylation patterns. While we investigated CpG methylation, there is increasing evidence for a potential functional role of non‐CpG methylation (Laine et al., 2016), which demands further research.

## CONFLICT OF INTERESTS

The authors declare no conflict of interest.

## Data Availability

RRBS and RNA‐seq raw reads are deposited in NCBI’s Sequence Read Archive (BioProject PRJNA314210).
